# Localized phosphoinositide metabolism regulates STIM1/ORAI1 fast inactivation

**DOI:** 10.1016/j.isci.2026.115543

**Published:** 2026-03-31

**Authors:** Ning Dai, Shawn M. Lamothe, Jody Groenendyk, Nicolas Touret, Harley T. Kurata, Marek Michalak

**Affiliations:** 1 Department of Biochemistry, University of Alberta, Edmonton, AB T6G 2H7, Canada; 2 Department of Pharmacology, University of Alberta, Edmonton, AB T6G 2H7, Canada

**Keywords:** Pharmacology, Biochemistry, Molecular biology

## Abstract

Store-operated Ca^2+^ entry (SOCE) is central for maintaining cellular Ca^2+^ homeostasis, and it is initiated by the depletion of Ca^2+^ in the endoplasmic reticulum (ER) and activation of stromal interaction molecule 1 (STIM1). STIM1 acts as an ER Ca^2+^ sensor and engages with plasma membrane ORAI1 channels to facilitate ORAI1 activation and Ca^2+^ influx. Here, we found that STIM1 forms a complex with myotubularin-related protein 7 (MTMR7) to regulate ORAI1 inactivation during prolonged Ca^2+^ entry. MTMR7 alters plasma membrane PI(3,5)P2 and PI(4,5)P2 levels, increasing ORAI1 inactivation and decreasing SOCE. Loss of catalytic phosphatase function of MTMR7 weakens ORAI1 inactivation and enhances SOCE activity, while the disruption of MTMR7 and STIM1 association retains ORAI1 inactivation. The MTMR7/STIM1 complex positions MTMR7 at ER-plasma membrane contact sites to fine-tune lipid signaling, prevent premature STIM1 activation, and modify ORAI1 inactivation. These findings provide insight into novel modes of regulation of ORAI1 inactivation by phosphoinositides (PIPs).

## Highlights

• MTMR7 phosphoinositide phosphatase forms a complex with stromal interaction molecule 1 (STIM1)
• MTMR7/STIM1 complex localizes phosphoinositide hydrolysis near ORAI1/STIM1 channels
• MTMR7/STIM1 complex controls ORAI1/STIM1 inactivation and store-operated Ca^2+^ entry (SOCE) termination

## Graphical abstract

**Figure:**
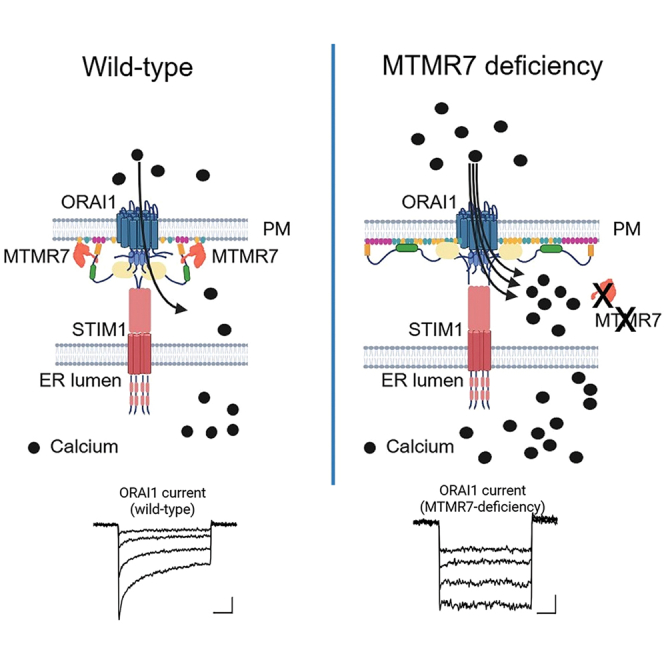

## Introduction

Store-operated Ca^2+^ entry (SOCE) is a fundamental mechanism to replenish intracellular Ca^2+^ stores and sustain long-term Ca^2+^ signaling.^1^ SOCE is activated in response to the depletion of Ca^2+^ from the endoplasmic reticulum (ER), the primary intracellular Ca^2+^ reservoir.^2^ This process involves two key molecular players: stromal interaction molecule (STIM) proteins, which function as ER Ca^2+^ sensors, and ORAI1 proteins, which form Ca^2+^-selective channels in the plasma membrane.^3^^,^^4^^,^^5^^,^^6^ Among the STIM family, STIM1 plays a critical role in initiating SOCE.^6^ It consists of an N-terminal ER-luminal domain that senses ER Ca^2+^ levels, a single-pass transmembrane domain, and a cytosolic C-terminal region responsible for transmitting activation signals.^7^ The ER-luminal domain contains EF-hand motifs, which bind Ca^2+^ and sense Ca^2+^ depletion in the ER. The cytosolic portion of STIM1 contains several key regions, including the STIM1-ORAI1 activating region (SOAR), the inactivation domain (ID), and the polybasic lysine-rich domain (K domain), all of which coordinate the activation and regulation of SOCE.^7^^,^^8^^,^^9^^,^^10^ The ID mediates interactions between the STIM1 inhibitor SOCE-associated regulatory factor (SARAF) and the SOAR domain to regulate ORAI1 channel function.^11^^,^^12^ Upon ER Ca^2+^ depletion, STIM1 undergoes a conformational change that exposes its SOAR domain, initiating translocation to ER-plasma membrane junctions/contact sites.^13^^,^^14^^,^^15^^,^^16^ At these sites, the K domain of STIM1 interacts with phosphatidylinositol 4,5-bisphosphate [PI(4,5)P2] on the cytosolic face of the plasma membrane, anchoring STIM1 in close proximity to its ORAI1 target.^12^^,^^17^ The SOAR domain of STIM1 then binds directly to ORAI1, triggering its activation and allowing extracellular Ca^2+^ to enter the cytoplasm.^10^^,^^18^^,^^19^ This increase in cytosolic Ca^2+^ allows sarco/ER Ca^2+^-ATPase (SERCA) to pump Ca^2+^ back into the ER lumen, replenishing ER Ca^2+^ stores and restoring homeostasis.^20^^,^^21^ This tightly regulated mechanism ensures cellular homeostasis and proper physiological function.

One of the initial driving forces for STIM1 translocation to the ER-plasma membrane contact sites is association with plasma membrane phosphoinositides (PIPs).^10^^,^^11^^,^^22^ We recently identified that myotubularin-related protein 7 (MTMR7), an active dual-specificity phosphatase that primarily dephosphorylates phosphatidylinositol 3-phosphate [PI(3)P] and Ins(1,3)P2, the cleaved inositide that is released from PI(3)P,^23^ forms a complex with STIM1.^24^ The MTMR family, consisting of 15 members, namely MTM1, MTMR1 to MTMR14, plays roles in phosphoinositide metabolism and cellular signaling.^25^^,^^26^^,^^27^^,^^28^ Based on the confirmation of MTMR7 phosphoinositide phosphatase activity, together with STIM1 phosphoinositide binding, we hypothesized that the MTMR7/STIM1 complex could modulate SOCE activity. Here we report that MTMR7/STIM1 complex positions the lipid phosphatase near STIM1/ORAI1 contact sites, which aligns its enzymatic activity to regulate local lipid signaling, inhibit early STIM1 activation, and modify ORAI1 inactivation. These findings provide insight into Ca^2+^ regulation by MTMR7, along with novel modes of regulation of ORAI1 inactivation by PIPs.

## Results

### MTMR7 is a STIM1 binding protein

**Figure 1:**
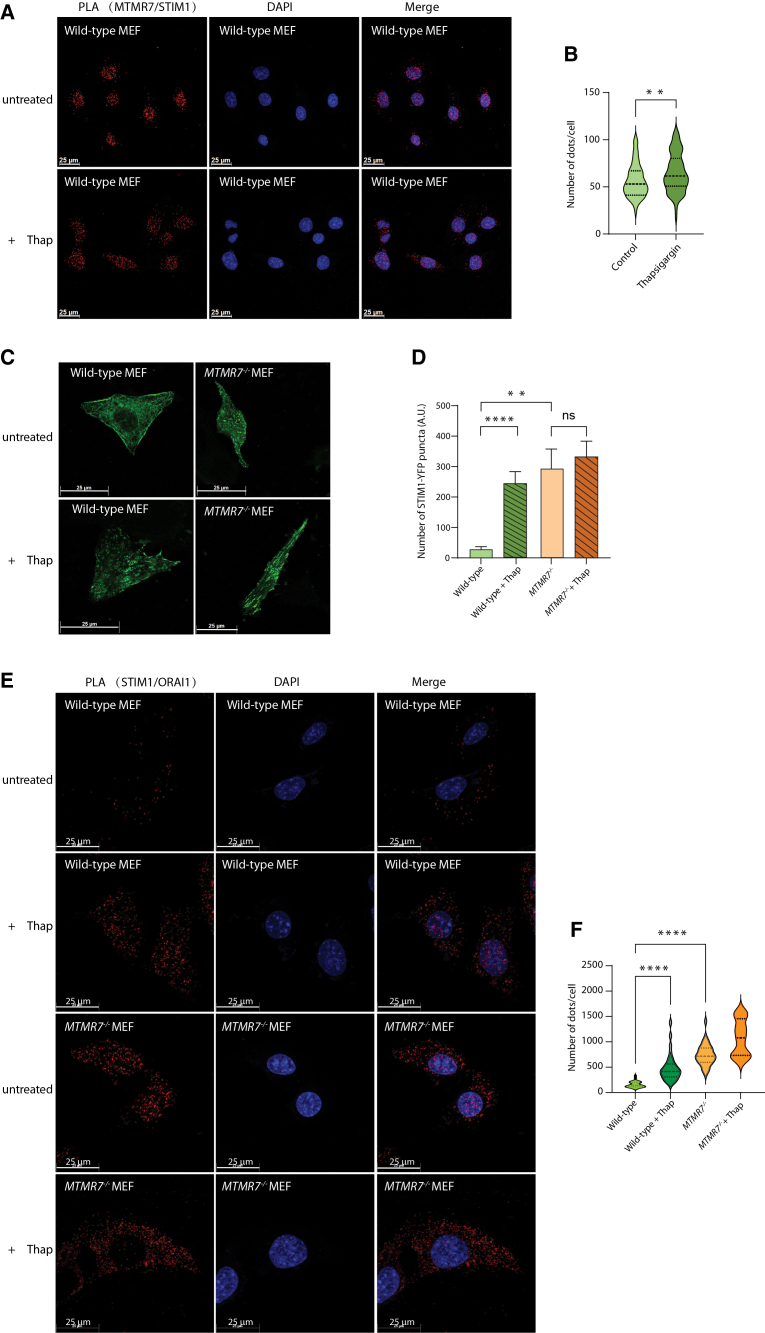
MTMR7 interacts with STIM1 (A) Proximity ligation assay detecting MTMR7 and STIM1 complex in wild-type cells alone (upper panel) and cells treated with thapsigargin (*+Thap*) (lower panel). Left: red color indicates MTMR7-STIM1 close proximity signals; middle: DAPI indicates nuclei; right: merge of left and middle panels. Maximum projections of Z-stacked images are shown. (B) Quantification of proximity ligation assay signal per cell, comparing untreated and thapsigargin-treated conditions. *n* = 80. ∗∗*p* < 0.01. (C) Confocal images of STIM1 puncta formation at the ER-plasma membrane in wild-type cells and *MTMR7*^−/−^ cells expressing STIM1-YFP in the absence (*untreated*) or 5 min after the addition of thapsigargin (*+Thap*). Scale bars, 25 μm. The figure displays the maximum projection of z stack images, with the entire cell compressed into a single 2D image. (D) Quantification of STIM1 puncta in wild-type and *MTMR7*^−/−^ cells in the absence (*untreated*) or presence of thapsigargin (*+Thap*). *n* = 15. ∗∗*p* < 0.01 and ∗∗∗∗*p* < 0.0001. ns, not significant. (E) Proximity ligation assay detection of STIM1 and ORAI1 in wild-type and *MTMR7*^−/−^ cells in the absence (*untreated*) or presence of thapsigargin (*+Thap*). (F) Quantification of proximity ligation assay signals in wild-type and *MTMR7*^−/−^ cells in the absence (*untreated*) or presence of thapsigargin (*+Thap*). *n* = 90. ∗∗∗∗*p* < 0.0001. Data are represented as mean ± SEM.

MTMR7 is a phosphoinositide phosphatase recently identified as a novel STIM1 binding protein.^23^ To further validate MTMR7/STIM1 complex formation, we used a Duolink proximity ligation assay to visualize close contacts between MTMR7 and STIM1 (Figures 1A and 1B). For Duolink proximity ligation analysis, we used anti-MTMR7 and anti-STIM1 antibodies and showed that the interaction between MTMR7 and STIM1 occurred in MEF cells expressing endogenous proteins (Figure 1A). Interestingly, thapsigargin-induced Ca^2+^ depletion of ER stores resulted in a modest increase in Duolink proximity ligation signal between MTMR7 and STIM1 (Figures 1A and 1B), indicating association between MTMR7 and STIM1 before and after ER Ca^2+^ depletion.

Then we asked how the formation of the MTMR7/STIM1 complex influences STIM1 and SOCE activation. To address this question, we used MTMR7-deficient (*MTMR7*^−/−^) cells to investigate whether STIM1 oligomerization (puncta formation) was influenced by MTMR7. Wild-type (WT) and *MTMR7*^−/−^ cells were transfected with a STIM1-YFP expression vector followed by the activation of SOCE by thapsigargin-induced ER Ca^2+^ depletion (Figure 1C). As expected, in WT cells, STIM1 displayed an ER-like distribution under basal conditions and formed puncta after 5 min treatment with thapsigargin (Figure 1C). In contrast, under basal conditions, *MTMR7*^−/−^ cells exhibited spontaneous STIM1 puncta formation in the absence of store depletion (Figures 1C and 1D). Next, we used Duolink proximity ligation to investigate whether STIM1 oligomers (puncta) seen in *MTMR7*^−/−^ cells (Figure 1C) resulted in STIM1/ORAI1 contact formation. *MTMR7*^−/−^ cells showed a higher number of Duolink positive STIM1/ORAI1 complexes compared to WT cells (Figures 1E and 1F) consistent with the increased STIM1-YFP puncta seen in *MTMR7*^−/−^ cells (Figures 1C and 1D). As expected, the addition of thapsigargin to activate SOCE led to an increase in Duolink positive (STIM1/ORAI1) spots in both WT and *MTMR7*^−/−^ cells (Figures 1E and 1F). Taken together, we concluded that MTMR7 interacts with STIM1 and may suppress its oligomerization and association with ORAI1, whereas MTMR7 deficiency was permissive for STIM1 oligomerization and association with ORAI1 (both prerequisites for SOCE). Importantly, we discovered that there was increased STIM1/ORAI1 complex formation in *MTMR7*^−/−^ cells even in the absence of ER Ca^2+^ depletion.

### MTMR7 deficiency leads to increased cytosolic and ER Ca^2+^

**Figure 2:**
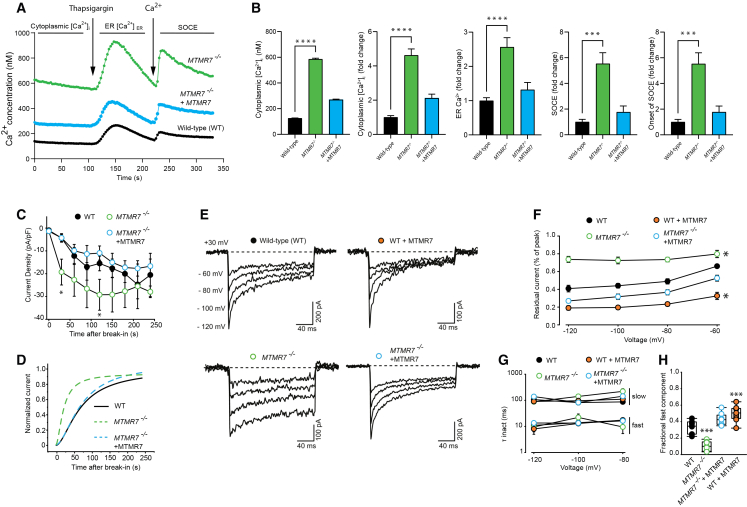
Delayed inactivation of ORAI1/STIM1 current in the absence of MTMR7 (A) Live-cell cytosolic Ca^2+^ concentration increase ([Ca^2+^]_i_) was monitored using Fura-2 AM. (B) Quantification of Ca^2+^ responses shown in Figure 2A *n* = 10, represents the number of independent experiments. ∗∗∗*p* < 0.01 and ∗∗∗∗*p* < 0.0001. (C) Current amplitudes from a −100-mV hyperpolarizing pulse for 200 ms were collected in 15–30 s intervals following whole-cell break-in up to 4 min. A nonparametric Kruskal-Wallis ANOVA on ranks followed by a multiple comparison (Dunn’s) post hoc test was used to compare between groups at the 30 s and 240 s time points. [∗*p* < 0.05; *MTMR7*^−/−^ vs. WT (ctrl)]. (ns = not significant). (D) Data from Figure 2C was normalized to the minimum and maximum peak and the rate of current onset was measured by fitting the data points at each time interval with a non-linear logistic equation (average time to 50% activation (s) ± SEM; WT = 68.5 ± 13.8; *MTMR7*^−/−^ = 20.9 ± 5.5; *MTMR7*^−/−^ + MTMR7 = 71.4 ± 19.3). The line of best fit of normalized current over time for each group was displayed as a graph in panel D. A nonparametric Kruskal-Wallis ANOVA on ranks followed by a multiple comparison (Dunn’s) post hoc test was used to compare the average time to 50% activation between all groups. [*p* = 0.04; *MTMR7*^−/−^ vs. WT (ctrl)]. (n = 5–13 cells per group). (E) Representative whole-cell current traces of heterologous expression of ORAI1 and STIM1 in wild-type (*WT*) cells or *MTMR7*^−/−^ cells in the presence or absence of recombinant MTMR7. Cells were transfected in a 1:2:1 (ORAI1:STIM1:MTMR7) ratio. Co-transfection with soluble GFP was used to ensure a constant amount of plasmid DNA in the transfection mixture between each group. Currents were recorded in 20 mM Ca^2+^ during 200 ms hyperpolarizing test voltages (−60, −80, −100, −120 mV) from a 30-mV holding potential. (F) Extent of STIM1/ORAI1 Ca^2+^-dependent inactivation (CDI) summarized from recordings of each group shown in Figure 2E. Line and scatterplot summarizing the fraction of current remaining for each group, measured as the percent of peak current from the beginning and the end of the 200 ms hyperpolarizing steps. Each data point represents the mean ± SEM (*n* = 10 cells for each group). A one-way ANOVA followed by a Dunnett’s post hoc test was used to compare the residual current of all groups against the control (WT) (∗*p* < 0.05 at all test potentials). (G) Fast and slow time constants (τ inact) from biexponential fits are plotted against test potentials (−80, −100, and −120 mV). Each data point represents the mean ± SEM. (H) The fractional fast component of the inactivating current at the −120-mV test pulse in all groups was displayed as boxplots. A one-way ANOVA followed by a Tukey’s post hoc test was used to compare all groups. (∗∗∗*p* < 0.001 vs. WT Ctrl). Data are represented as mean ± SEM.

Next, we used the Fura-2 AM fluorescent Ca^2+^ indicator to determine whether MTMR7 binding to STIM1 influenced cellular Ca^2+^ homeostasis and activation of SOCE. WT and *MTMR7*^−/−^ cells were loaded with Fura-2 AM followed by monitoring of cytosolic Ca^2+^. As expected, in resting WT cells, cytosolic Ca^2+^ concentration was near 150 nM (Figure 2A). However, cytosolic Ca^2+^ concentration in the *MTMR7*^−/−^ cells reached levels over 600 nM (Figure 2A) likely due to enhanced STIM1/ORAI1-mediated Ca^2+^ entry. The expression of MTMR7 in the *MTMR7*^−/−^ cells reversed this effect and significantly lowered cytosolic Ca^2+^ concentration close to WT levels (Figures 2A and 2B), demonstrating dependence on MTMR7. The fact that expressing MTMR7 in *MTMR7*^−/−^ cells only partially restores elevated steady-state cytoplasmic Ca^2+^ likely indicates that long-term loss of MTMR7 leads to compensatory changes. This type of partial recovery has also been observed with other ER-plasma membrane Ca^2+^ regulators, highlighting the complexity of basal Ca^2+^ regulation.^29^^,^^30^ Next, cytosolic Ca^2+^ concentration was monitored following the addition of thapsigargin to evaluate the amount of releasable ER Ca^2+^. The addition of thapsigargin led to markedly elevated Ca^2+^ release in *MTMR7*^−/−^ cells compared to WT cells (Figures 2A and 2B), indicating increased ER luminal thapsigargin-sensitive Ca^2+^ stores in *MTMR7*^−/−^ cells. However, it cannot be excluded that the apparent change in the thapsigargin-sensitive ER Ca^2+^ content may reflect long-term cellular adaptations to MTMR7 deficiency. Again, the expression of MTMR7 in the *MTMR7*^−/−^ cells reduced thapsigargin-induced Ca^2+^ release from ER (Figures 2A and 2B). Next, we monitored SOCE activity by the addition of extracellular Ca^2+^ to WT and *MTMR7*^−/−^ cells previously treated with thapsigargin to deplete ER Ca^2+^. SOCE-mediated Ca^2+^ influx was increased in *MTMR7*^−/−^ cells and characterized by a steeper rise to the Ca^2+^ peak (Figures 2A and 2B), indicative of a rapid influx of extracellular Ca^2+^. Again, this was sensitive to the expression of recombinant MTMR7 in *MTMR7*^−/−^ cells (Figures 2A and 2B). We concluded that MTMR7 deficiency resulted in increased cytosolic Ca^2+^ concentration, thapsigargin-induced releasable Ca^2+^ pool, and did not interfere with Ca^2+^ influx via SOCE.

Upon ER Ca^2+^ depletion, STIM1 oligomerizes (puncta formation) and extends to ER-plasma membrane contact sites, where it forms a complex with ORAI1.^9^^,^^31^^,^^32^^,^^33^ Since MTMR7 is a STIM1-interacting protein (Figure 1),^23^ we hypothesized that MTMR7-mediated changes in cytosolic and ER luminal Ca^2+^ could arise from alterations in SOCE. We directly measured SOCE using whole-cell patch clamp with ER Ca^2+^ depletion (thapsigargin), intracellular Ca^2+^ chelators (e.g., EGTA and BAPTA), and high extracellular Ca^2+^, to promote STIM1/ORAI1 activation and facilitate the detection of current.^34^^,^^35^ WT or *MTMR7*^−/−^ cells were transfected with STIM1 and ORAI1 expression vectors along with either GFP or MTMR7-GFP and cultured for 24–48 h before recording. Currents were elicited with a −100 mV hyperpolarizing pulse and current density was plotted every 30 s for 4 min (Figure 2C). The resulting STIM1/ORAI1 currents in *MTMR7*^−/−^ cells exhibited rapid development of current upon whole-cell break-in (time to 50% activation of 20.9 s) (Figure 2D). In contrast, STIM1/ORAI1 currents in WT cells exhibited a lag in current initiation and a slower rate to maximal sustained current (Figure 2D). The expression of recombinant MTMR7 in the *MTMR7*^−/−^ cells mimicked the delayed current onset observed in WT cells [time to 50% activation of 71.4 s in *MTMR7*^−/−^ + MTMR7 cells versus 68.5 s in WT cells] (Figure 2D). These findings indicate the inhibition of SOCE initiation by MTMR7.

To further investigate molecular events associated with altered STIM1/ORAI1 function in *MTMR7*^−/−^ cells, we explored hallmark features of STIM1/ORAI1 currents. ORAI1 channels undergo fast Ca^2+^-dependent inactivation (CDI) arising from rapid Ca^2+^ feedback through the pore.^35^^,^^36^ To measure CDI, we recorded currents from WT and *MTMR7*^−/−^ cells, with 200 ms voltage steps between −60 and −120 mV pulses as previously described.^37^^,^^38^ A constant STIM1/ORAI1 transfection ratio (2:1) was maintained throughout the study, as the extent of CDI depends on the expression ratio of these two proteins.^34^^,^^39^ STIM1/ORAI1 currents exhibited typical rapid biexponential inactivation in WT cells. However, in *MTMR7*^−/−^ cells, the onset of inactivation was markedly weaker (Figures 2E and 2F), leading to a much larger residual current at the end of each voltage pulse (Figure 2E, normalized data).

Prominent CDI was rescued in *MTMR7*^−/−^ cells upon the co-transfection of the MTMR7 expression vector with STIM1 and ORAI1 expression vectors. Further, MTMR7 overexpression in WT cells increased the extent of CDI (Figures 2E and 2F). The peak current-voltage relationships for STIM1/ORAI1 in WT versus *MTMR7*^−/−^ cells after 3 min following break-in (Figure 2C) and the kinetics of the fast and slow components of the biexponential inactivation (Figure 2G) were virtually indistinguishable and not affected by co-transfection with the MTMR7 expression vector. However, the relative contribution of the two components was different between groups, with the inactivation current in *MTMR7*^−/−^ cells displaying less of a fast component compared to WT cells (Figure 2H). Currents exhibited comparable inward rectification in both cell lines (independent of MTMR7 expression) with a strong positive reversal potential (∼+60 to 80 mV), indicative of high Ca^2+^ selectivity (Figure S1). Taken together, these findings demonstrate that MTMR7 regulates ORAI1 gating, and its absence facilitated assembly of the STIM1/ORAI1 complex, even in the absence of ER Ca^2+^ depletion. It is noteworthy to mention that despite observing STIM1/ORAI1 interaction in *MTMR7*^−/−^ cells in the absence of thapsigargin (Figure 1), minimal currents were observed immediately after whole-cell break-in. Therefore, we cannot conclude that the STIM1/ORAI1 channel was in a fully constitutively active state in *MTMR7*^−/−^ cells, activating independently from ER Ca^2+^-depletion. Nevertheless, the current onset and time to peak current were markedly faster in *MTMR7*^−/−^ compared to WT, upon adequate diffusion of Cs-BAPTA into the cell, indicating a change in STIM1/ORAI1 assembly and activity. Due to the use of a high concentration of a fast-acting chelator (20 mM BAPTA) and thapsigargin, in his study, we have not quantified the effect on slow calcium-dependent inactivation (SCDI).

### MTMR7 catalytic activity, rather than interaction with STIM1, regulates STIM1/ORAI1

**Figure 3:**
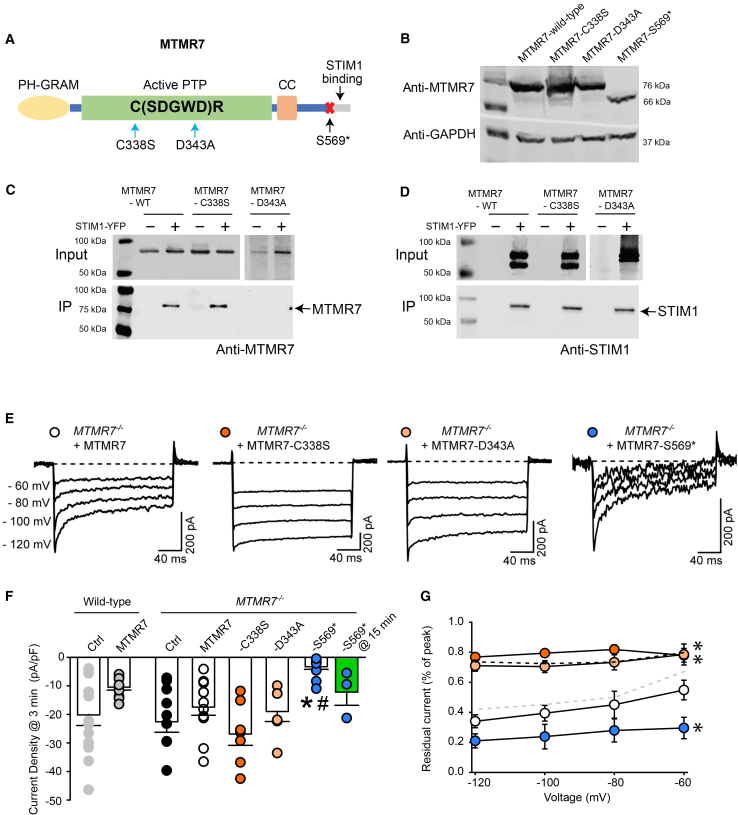
Catalytically active MTMR7 is essential for STIM1/ORAI1 activity (A) Schematic representation of the MTMR7 protein domains. The PH-GRAM domain is shown in yellow, the catalytic protein tyrosine phosphatase (PTP) domain in green, and the coiled-coil (CC) domain in pink. The location of the STIM1 binding region at the C-terminus of MTMR7 is indicated in the figure. The location of the stop codon introduced at the N-terminal amino acid residues of the STIM1 interaction site (S569∗) is indicated by a red X, the location of C338S and D343A mutations in the catalytic PTP domain is indicated by blue arrows. (B) Immunoblot analysis of wild-type and MTMR7 mutants expressed in HEK293T cells. Anti-GAPDH was used as a loading control. (C and D) Co-immunoprecipitation of MTMR7 and STIM1 in HEK293T cells co-transfected with STIM1-YFP and either MTMR7, MTMR-C338S, or MTMR-D343A mutants. Immunoprecipitation was performed using GFP-Trap agarose beads, followed by immunoblotting with anti MTMR7 (*C*) and anti STIM1 (*D*) antibodies. (E) Representative whole-cell current traces of heterologously expressed ORAI1 and STIM1, along with MTRM7 or MTMR7 mutants MTMR7-C338S, MTMR7-D343A, and MTMR7-S569∗ in *MTMR7*^−/−^ cells. Cells were transfected in a 1:2:1 (ORAI1:STIM1:MTMR7 or MTMR7 mutants) ratio. Currents were recorded in 20 mM Ca^2+^ during 200 ms hyperpolarizing test voltages (−60, −80, −100, and −120 mV) from a 30-mV holding potential. (F) Current density (pA/pF) analysis of current from a −100-mV hyperpolarizing pulse at 3 min following whole-cell break-in (white bars; n = 6–13 for each group). Green bar denotes the current density measured from a −100-mV pulse at 15 min following whole-cell break-in (*n* = 3). A nonparametric Kruskal-Wallis ANOVA on ranks test, followed by a multiple comparison (Dunn’s) post hoc test, was used to compare each group against either the control (*Ctrl*) condition in wild-type or *MTMR7*^−/−^ MEF cells (∗*p* < 0.001 vs. wild-type, #*p* < 0.01 vs. *MTMR7*^−/−^). (G) Extent of STIM1/ORAI1 CDI from recordings of each group shown in Figure 3F. Line and scatterplot summarizing the fraction of current remaining for each group, measured as the percent of peak current from the beginning and the end of the 200 ms hyperpolarizing steps. Each data point represents the mean ± SEM (*n* = 6–15 cells for each group). For comparison, the data from wild-type or *MTMR7*^−/−^ cells were superimposed from Figure 2D in gray and black dashed lines, respectively. A one-way ANOVA followed by a Dunnett’s post hoc test was used to compare the residual current of all groups against the control (WT) (∗*p* < 0.05 at all test potentials). Data are represented as mean ± SEM.

MTMR7 is a phosphoinositide phosphatase,^24^^,^^40^ and STIM1-mediated activation of ORAI1 channel depends initially on STIM1’s interaction with PIPs on the plasma membrane.^12^^,^^17^ We asked, therefore, whether MTMR7 phosphatase activity and/or binding to STIM1 were required for MTMR7-dependent effects on STIM1/ORAI1 activity. Catalytically active myotubularin has a highly conserved “C(X)_5_R” catalytic site with a sequence of “CSDGWDR” (Figure 3A).^40^^,^^41^ Many studies have mutated the first cysteine to serine to create phosphatase-inactive mutations for MTMRs.^42^^,^^43^^,^^44^^,^^45^^,^^46^^,^^47^ A mutation at the second aspartic acid to alanine is also a common inactivation mutation that disrupts the hydrogen bond between aspartic acid and scissile oxygens.^48^ Therefore, we generated MTMR7 phosphatase inactive mutants designated MTMR7-C338S and MTMR7-D343A, along with a C-terminal deletion MTMR7 mutant (MTMR7-S569∗) that removes the previously identified STIM1 binding region but preserves the catalytic site (Figure 3A). The C338S and D343A mutants abolished catalytic activity with C338S having minimal impact on MTMR7 PTP structure.^49^ However, the D343A mutant removes a charged residue, and the absence of aspartate prevents hydrolysis and substrate release.^50^ This results in a disrupted hydrogen bonding, predicting possible conformational changes in the catalytic domain.

*MTMR7*^−/−^ cells were transfected with expression vectors encoding MTMR7-C338S, MTMR7-D343A, or MTMR7-S569∗ followed by immunoblot analysis (Figure 3B). Expression levels of MTMR7-D343A or MTMR7-S569∗ mutants were consistently lower than WT MTMR7 and MTMR7-C338S mutant (Figure 3B), and this was not affected by the proteasome inhibitor MG132, indicating that the protein was not being degraded. Next, we carried out immunoprecipitation analysis using GFP-Trap agarose beads to determine whether MTMR7 mutants could interact with STIM1-YFP. As previously reported, WT MTMR7 was efficiently immunoprecipitated with STIM1-YFP, indicating complex formation between MTMR7 and STIM1 (Figures 3C and 3D).^23^ Next, we tested for complex formation between STIM1 and MTMR7-C338S and MTMR7-D343A phosphatase inactive mutants. Immunoprecipitation analysis demonstrated the association of catalytically inactive MTMR7-C338S and STIM1 (Figures 3C and 3D). Surprisingly, the MTMR7-D343A did not form a complex with STIM1-YFP as tested by immunoprecipitation (Figures 3C and 3D). This is likely due to disrupted hydrogen bonding (absence of aspartate) and could lead to conformational changes in the catalytic core of MTMR7, which may alter the orientation of its C-terminal domain and affect the formation of the MTMR7/STIM1 complex.

Next, we examined the effect of MTMR7 mutants on STIM1/ORAI1 channel inactivation. *MTMR7*^−/−^ cells were transfected with WT or MTMR7 mutants (MTMR7-C338S, MTMR7-D343A or MTMR7-S569∗), and STIM1 and ORAI1 expression vectors. Consistent with Figure 2, WT MTMR7 co-expression promoted CDI of STIM1/ORAI1, however, the catalytically inactive mutants (MTMR7-C338S, MTMR7-D343A) lead to very little inactivation (Figures 3E and 3G), comparable to STIM1/ORAI1 activity in *MTMR7*^−/−^ cells (Figure 2C). Interestingly, the truncated (but catalytically active) MTMR7-S569∗ mutant exhibited extensive CDI (Figures 3E and 3G). Notably, MTMR7-S569∗ led to extremely small STIM1/ORAI1 currents that were very slow to develop, requiring approximately 15 min after whole-cell break-in to develop appreciable ORAI1 current (Figures 3E and 3F). This current development was much slower than observed in cells expressing WT MTMR7 (Figure 2C). The current-voltage relationship and inward rectification properties for STIM1/ORAI1 co-expressed with the MTMR7 mutations were not different from WT MTMR7 (Figure S1). These results indicate that the catalytic activity of MTMR7 influences CDI of STIM1/ORAI1, but that direct interactions with STIM1 were not essential for the regulation of STIM1/ORAI1 inactivation. In fact, the disruption of MTMR7 association with STIM1 caused even more severe functional outcomes in terms of STIM1/ORAI1 gating. It should be noted that C338S and D343A represent different catalytic mutants: C338S interacted with STIM1, but D343A did not, potentially due to an altered conformation. The S569∗ truncation resulted in slow, small ORAI1 currents with robust CDI, suggesting catalytic activity alone can support CDI without STIM1 binding, though additionally, an S569∗ mis-localization or altered protein stability may also affect PIP levels and SOCE.

**Figure 4:**
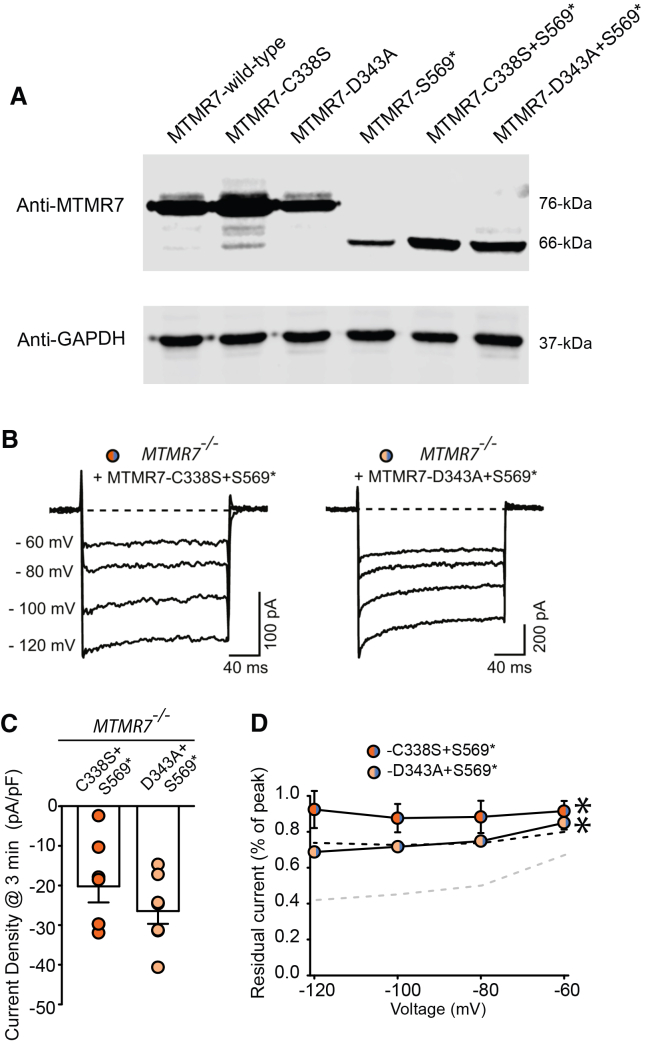
MTMR7 double mutants and ORAI1 inactivation (A) Immunoblot analysis of HEK293T cells expressing MTMR7 and MTMR7 mutants (MTMR7-C338S, MTMR7-D343A, MTMR7-C338S + S569∗, and MTMR7-D343A + S569∗). (B) Representative whole-cell current traces of heterologously expressed ORAI1 and STIM1, along with MTMR7 double mutants; MTMR7-C338S + S569∗ or MTMR7-D343A + S569∗ in *MTMR7*^−/−^ cells. Cells were transfected in a 1:2:1 (ORAI1:STIM1:MTMR7 double mutants) ratio. Currents were recorded in 20 mM Ca^2+^ during 200 ms hyperpolarizing test voltages (−60, −80, −100, −120 mV) from a 30-mV holding potential. (C) Current density (pA/pF) analysis of current from a −100-mV hyperpolarizing pulse at 3 min following whole-cell break-in (white bars; *n* = 7 for each group). (D) Extent of STIM1/ORAI1 CDI from recordings of each group shown in Figure 3F. Line and scatterplot summarizing the fraction of current remaining for each group, measured as the percent of peak current from the beginning and the end of the 200 ms hyperpolarizing steps. Each data point represents the mean ± SEM (n = 8–9 cells for each group). For comparison, the data from wild-type MEF or *MTMR7*^−/−^ cells were superimposed from Figure 2D in gray and black dashed lines, respectively. A one-way ANOVA followed by a Dunnett’s post hoc test was used to compare the residual current of all groups against the control (WT) (∗*p* < 0.05 at all test potentials). Data are represented as mean ± SEM.

To further investigate the role of the catalytic activity of MTMR7 versus MTMR7-STIM1 complex formation in the regulation of STIM1/ORAI1 activity, we generated double mutants with mixed catalytic site mutations and the S569∗ C-terminal deletion MTMR7 (MTMR7-C338S/S569∗ and MTMR7-D343A/S569∗) (Figure 4A). Patch clamp analysis of STIM1/ORAI1 activity was carried out in *MTMR7*^−/−^ MEF cells expressing STIM1/ORAI1 along with each double mutant (Figure 4B). Unlike the currents for the MTMR7-S569∗ mutant, both double mutants produced sizable currents shortly after whole-cell break-in, comparable to the STIM1/ORAI1 recordings in *MTMR7*^−/−^ cells (Figure 4C). Both double mutants also exhibited weak CDI, similar to the individual catalytic site mutants (Figures 4B and 4D) indicating that catalytic activity of MTMR7 was the most important feature for control of STIM1/ORAI1 CDI.

### MTMR7 influences PIPs adjacent to STIM1

**Figure 5:**
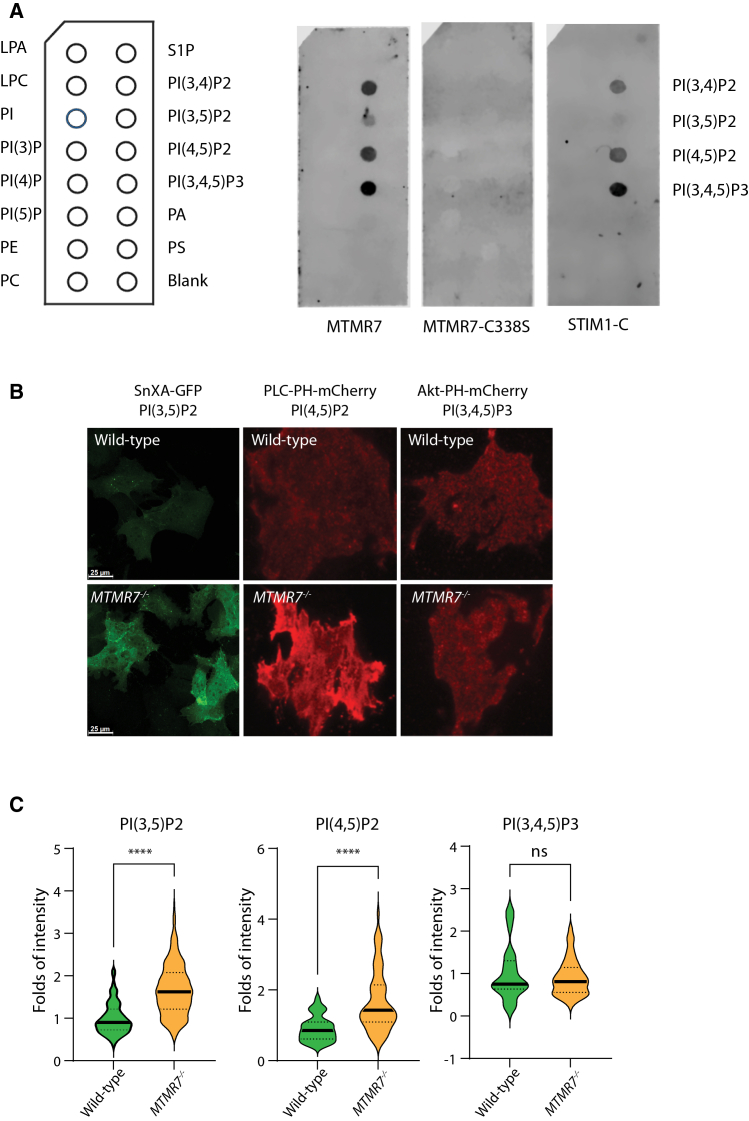
Phosphoinositide binding and plasma membrane phosphoinositide in the absence of MTMR7 (A) Lipid-protein overlay assay using GST-tagged MTMR7, MTMR7-C338S, and the C-terminal region of STIM1 (STIM1-C). Nitrocellulose membranes spotted with indicated phospholipids (*left*) were incubated with purified proteins GST-tagged MTMR7, MTMR7-C338S, and STIM1-C. *n* = 3. (B) Confocal microscope images of wild-type and *MTMR7*^−/−^ cells expressing phosphoinositide-specific fluorescent probes: SnxA-GFP for PI(3,5)P2, PLC-PH-mCherry for PI(4,5)P2, and Akt-PH-mCherry for PI(3,4,5)P3. Cells were imaged under resting conditions. Scale bars, 25 μm. (C) Quantification of fluorescence intensity for each phosphoinositide probe shown in Figure 5B. *MTMR7*^−/−^ cells exhibited significantly increased levels of PI(3,5)P2 and PI(4,5)P2 compared to wild-type cells, while PI(3,4,5)P3 levels remained unchanged. Data represent fold change in mean fluorescence intensity per cell. ∗∗∗∗*p* < 0.0001; ns, not significant. Data are represented as mean ± SEM.

Upon ER Ca^2+^ depletion, STIM1 oligomers interact with PI(4)P and PI(4,5)P2 prior to binding to the ORAI1 and activation of ORAI1-dependent Ca^2+^ entry.^10^ We tested for MTMR7 and STIM1 binding to PIPs using a PIP strip membrane assay. The protein-lipid assay indicated a strong interaction between MTMR7 and PI(3,4)P2, PI(4,5)P2 and PI(3,4,5)P3 and relatively weak binding signal to PI(3,5)P2 (Figure 5A). Association with PI(4,5)P2 (Figure 5A) was clear but unexpected, as this phosphoinositide is not recognized by other MTMRs.^40^^,^^41^ We also tested for PIP binding of the purified cytoplasmic C-terminal domain of STIM1. In agreement with a previous report,^10^ the STIM1 C-terminal region bound to PI(3,4)P2, PI(3,5)P2, PI(4,5)P2, and PI(3,4,5)P3 with the weakest signal for PI(3,5)P2 (Figure 5A). Collectively, the findings above show that both MTMR7 and STIM1 cytoplasmic domain interact directly with the same set of PIPs, with the strongest interaction signal observed for PI(3,4)P2, PI(4,5)P2, and PI(3,4,5)P3.

Next, we used total internal reflection fluorescence (TIRF) microscopy to examine whether MTMR7 influenced the abundance of plasma membrane PIPs (Figure 5B). We used PIP reporters specifically for PI(4,5)P2 (the PH domain of phospholipase C tagged with mCherry), PI(3,5)P2 (the highly selective binding protein SnxA tagged with GFP), and PI(3,4,5)P3 [the PH domain of Akt (protein kinase B) tagged with mCherry]. TIRF analysis revealed a significant increase in PI(3,5)P2 and PI(4,5)P2 in *MTMR7*^−/−^ cells (Figures 5B and 5C) while PI(3,4,5)P3 remained unchanged (Figures 5B and 5C). Taken together, the above results indicate that MTMR7 and STIM1 form a complex and interact with an overlapping set of PIPs at the plasma membrane. These findings indicate that MTMR7 influences the levels of plasma membrane PIPs, such as PI(3,5)P2 and PI(4,5)P2. The increased concentration of these lipids in *MTMR7*^−/−^ cells is associated with changes in ORAI1 gating, which is dependent on the catalytic activity of MTMR7. Therefore, complex formation between MTMR7 and STIM1 provides a means for MTMR7 to be strategically located in the vicinity of the STIM1-ORAI1complex to regulate SOCE activity.

## Discussion

In this study, we identified previously unrecognized roles for MTMR7 in regulating SOCE dynamics and Ca^2+^ homeostasis. MTMR7, via its unique C-terminus, directly associates with STIM1,^23^ and prominently affects the inactivation of the ORAI1 channel and overall SOCE activity. Our findings identify MTMR7 as a novel modulator of ORAI1 inactivation, requiring its catalytic phosphatase function and influenced by a direct interaction with STIM1. We postulate that the MTMR7/STIM1 association, mediated by the C-terminus of MTMR7, anchors its catalytic activity in proximity to the STIM1/ORAI1 complex at ER-plasma membrane contact sites. This spatial positioning allows MTMR7 to locally regulate phosphoinositide metabolism and thereby influence the dynamics of STIM1/ORAI1 assembly, inactivation, and SOCE termination.

We uncovered that MTMR7 dynamically regulates SOCE activity and the plasma membrane PIPs. Given that SOCE activity is known to be controlled by PIPs, we propose that MTMR7 may affect the gating of the ORAI1 channel by modulating the plasma membrane abundance of phosphoinositide phospholipids. In this study, the specificity of MTMR7 for PI(3,5)P2 and PI(4,5)P2 is supported by results from binding assays and reporter intensity measurements; however, the potential involvement of other PIPs cannot be excluded. Catalytically active MTMR7, but not the C338S mutant, directly binds to multiple PIPs, including PI(3,5)P2, PI(4,5)P2, and PI(3,4,5)P3. This interaction is significant, given the established role of PI(4,5)P2 in anchoring STIM1 at the ER-plasma membrane to facilitate STIM1/ORAI1 coupling.^17^^,^^51^ We showed that MTMR7 influences PIPs [PI(3,5)P2 and PI(4,5)P2] abundance, at the plasma membrane, and depletion of these PIPs leads to altered STIM1/ORAI1 assembly (e.g., Figure 2D), and diminished SOCE activity (including accelerated inactivation). There is a persistent high abundance of PI(3,5)P2 and PI(4,5)P2 in *MTMR7*^−/−^ cells, likely prolonging STIM1 tethering and ORAI1 activation, and this explains the sustained Ca^2+^ entry and Ca^2+^ overload that we observed in *MTMR7*^−/−^ cells. Most importantly, we demonstrated that MTMR7 dynamically regulates ORAI1 gating properties and SOCE activity, and MTMR7 deficiency facilitates assembly of STIM1/ORAI1 complex, even in the absence of ER Ca^2+^ depletion. Although we cannot directly measure rates of STIM1/ORAI1 complex disassembly, an interesting possibility is that localized PIP dephosphorylation by MTMR7 contributes to disassembly of STIM1/ORAI1 and potentially other PIP-dependent proteins that influence the formation of ER-plasma membrane contact sites (e.g., GRAMD2a).^52^ It remains to be established whether MTMR7 impacts STIM1/ORAI1 signaling by the local depletion of certain PIPs at STIM1/ORAI1 contact sites or there is a broader change in PIP metabolism.

The exact mechanisms underlying ORAI1 inactivation and SOCE termination have yet to be fully elucidated. The ID regulatory region in STIM1 is involved in promoting the inactivation of ORAI1.^7^^,^^11^^,^^53^^,^^54^^,^^55^ Mutation or deletion of the ID of STIM1 abolishes CDI due to the disruption of the interactions between the STIM1 SOAR and ID and mono-PIPs.^10^^,^^11^^,^^37^^,^^53^^,^^54^ CDI of STIM1/ORAI1 current is also controlled by other modulators, including calmodulin^37^ and SARAF, an ER resident protein, which interacts with the C-terminus of STIM1.^11^^,^^12^ While we cannot rule out the influence of these regulators in our system, our results suggest that the mechanisms for the MTMR7 regulation of ORAI1 CDI arise from the hydrolysis of one or more PIPs, thereby altering the interaction of STIM1 with ORAI1 and regulating inactivation gating. In our study, CDI was prominently affected by the catalytic activity of MTMR7, indicating that high abundance of PIPs stabilizes the ID in a conformation that prevents the onset of CDI. It was also surprising that loss of direct MTMR7/STIM1 association in the MTMR7-S569∗ truncation mutant caused even more powerful suppression of STIM1/ORAI1, suggesting a gain-of-function effect of MTMR7-S569∗. This could arise from the mislocalization of MTMR7, leading to more widespread/unregulated effects on lipid levels throughout the cell, rather than at specific membrane contact sites, although our findings do not demonstrate this with certainty. Nevertheless, the powerful effects of the S569∗ mutant are eliminated in catalytic-deficient double mutants, indicating that S569∗ effects are likely caused by unregulated catalysis. Whether MTMR7 dephosphorylates mono- or bis-PIPs at membrane contact sites to modulate STIM1/ORAI1 interactions represents a compelling avenue for future research. Overall, we propose that in WT cells, MTMR7 binds to STIM1 and is recruited to the ER-plasma membrane contact sites, localizing its phosphatase activity, thereby affecting PI(4,5)P2 and PI(3,5)P2, and potentially other PIPs. Consequently, the catalytic activity of MTMR7 may impact STIM1 association with PIPs and consequent assembly with ORAI1, along with specific PIP-dependent effects on ORAI1 CDI.

MTMR7 is a STIM1 binding protein (this study)^23^ and, notably, the interaction site for STIM1 resides within a C-terminal intrinsically disordered region that is unique to MTMR7, distinguishing it from other MTMR family members.^56^ The C-terminal region also facilitates additional protein-protein interactions, underpinning the multivalent nature of MTMR7. Beyond its STIM1 interaction, MTMR7 heterodimerizes with MTMR9 and directly engages with PPARγ and RAS proteins through its coiled-coil domain.^57^^,^^58^ Binding of the unique MTMR7 C-terminal region to STIM1 helps target the phosphatase to specific subcellular compartments at ER-plasma membrane contact sites rich in lipids such as PI(3,5)P2 and PI(4,5)P2. This could create specificity and efficiency of Ca^2+^ signaling in terms of assembly and gating of STIM1/ORAI1 clusters.

**Figure 6:**
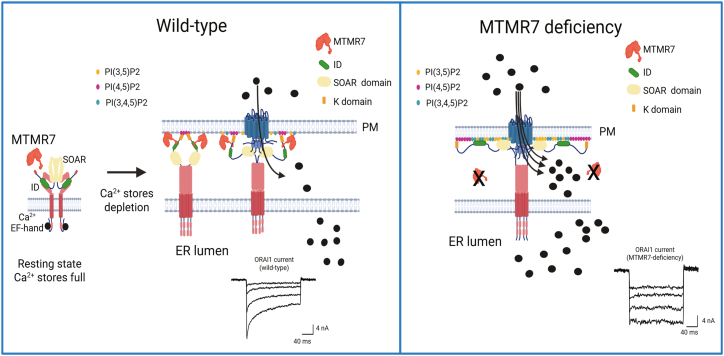
A schematic representation of the role of MTMR7 in the regulation of SOCE *Left*: In wild-type cells, MTMR7 forms a complex with STIM1, and consequently, MTMR7 is localized in close proximity to the STIM1/ORAI1 interaction site, generating hallmark inactivating ORAI1 currents. *Right:* In the absence of MTMR7 (*MTMR7-deficiency*), there is an increased abundance of PI(3,5)P2 and PI(4,5)P2 in the plasma membrane, resulting in a prolonged association of K-domain, SOAR domain, and ID of STIM1 and altered inactivation of SOCE, ultimately affecting ORAI1 gating properties and resulting in increased Ca^2+^ entry via ORAI1. Generated with BioRender. https://BioRender.com/8l56jxr.

Based on our findings, we propose the following role for MTMR7 in the regulation of SOCE (Figure 6). Under physiological conditions, MTMR7 is distributed in the cytosol and interacts with STIM1. Upon the depletion of ER Ca^2+^ stores, the STIM1 cytoplasmic C-terminus adopts an extended configuration, and MTMR7/STIM1 engages with ORAI1 channels at the ER-plasma membrane contact site. Ca^2+^ store depletion-induced conformational changes of the cytoplasmic C-terminus of STIM1 expose the SOAR, the ID and K domain of STIM1, enabling interaction with plasma membrane lipids including PI(4)P, PI(3,5)P2, PI(4,5)P2, and PI(3,4,5)P3,^10^^,^^12^^,^^13^^,^^17^^,^^18^^,^^59^ and subsequently, the SOAR domain interacts with ORAI1 to initiate Ca^2+^ entry. With respect to the initial PI(4,5)P2 binding, the K domain shows a preference for PI(4,5)P2 binding while the SOAR and ID prefer PI4P, PI(3,5)P2, and PI(3,4,5)P3 ^10^. In this study, we discovered that in the absence of MTMR7, there is increased abundance of plasma membrane PI(3,5)P2 and PI(4,5)P2, which could influence the initial contact of each of these domains (K, ID, SOAR) with the plasma membrane.^10^^,^^17^ This was also accompanied by impaired ORAI1 inactivation. Our results suggest that in *MTMR7*^−/−^ cells, increased abundance of PI(3,5)P2 and PI(4,5)P2 stabilized the STIM1 SOAR and ID, promoting the assembled STIM1/ORAI1 complex and preventing ORAI1 inactivation (Figure 6). Consequently, there is a prolonged activity of ORAI1 channels due to failure of the PIP bound ID to inactivate the channel.

In summary, our findings reveal a previously unrecognized role for MTMR7 cellular distribution and catalytic activity in influencing the inactivation of ORAI1 current during sustained Ca^2+^ entry, leading to SOCE termination. These findings underscore MTMR7 as a critical modulator of Ca^2+^ signaling and suggest that MTMR7 dysregulation may contribute to pathological conditions involving Ca^2+^ imbalance. MTMR7 is found in many human tissues, including the brain, gastrointestinal tract, kidney, liver, and testis, with its highest levels in the brain. MTMR7 is expressed across all major brain regions, such as the cerebral cortex, hippocampus, cerebellum, midbrain, and spinal cord. The MTMR7-SOCE signaling pathway may be particularly important in the central nervous system, where Ca^2+^ entry is crucial for neuronal excitability and synaptic communication.

### Limitations of the study

This study primarily employed loss-of-function strategies (MTMR7 knockout) to elucidate the role of MTMR7 in regulating PIPs and SOCE. However, some details about how MTMR7 impacts STIM1/ORAI1 signaling are still unclear, such as whether it causes the local depletion of certain PIPs at STIM1/ORAI1 junctions or leads to broader changes in PIP metabolism or membrane structure. Further research on the intracellular localization of MTMR7 and its mutants could clarify if protein mis-localization is linked to widespread or uncontrolled lipid changes within the cell. The investigation was restricted to the STIM1/MTMR7 complex, and the involvement of STIM2 in MTMR7-dependent signaling may need to be addressed.

## Resource availability

### Lead contact

Further information and requests for resources should be directed to and will be fulfilled by the lead contact. Marek Michalak. Email: marek.michalak@ualberta.ca.

### Materials availability

This study did not generate new unique reagents.

### Data and code availability

• The datasets generated during in this study are available from the lead contact upon reasonable request.
• Original western blot images have been deposited at https://osf.io/ujez7/ and are publicly available as of the date of publication. Microscopy data reported in this paper will be shared by the lead contact upon request.
• This paper does not report original code.

## Acknowledgments

This work was supported by a generous donation from the Kenneth and Sheelagh McCourt family, the 10.13039/501100018911University Hospital Foundation; the 10.13039/501100000024Canadian Institutes of Health Research grant MOP-15291; 10.13039/501100000038Natural Sciences and Engineering Research Council of Canada grant RGPIN-2019-04908. ND was a recipient of the China Council Scholarship. SML was funded by a Canadian Institutes of Health Research Post-Doctoral Fellowship. We also thank Kiara Smith, Dr. Strickfaden, and the Cell Imaging Center of the University of Alberta for their assistance with microscopy.

## Author contributions

N.D. designed experiments, analyzed data, and performed biochemical, cell biological, immunological analyses, wrote and edited the manuscript; S.M.L. designed experiments, analyzed data, and performed electrophysiological experiments and analysis, wrote and edited the manuscript; J.G. designed experiments, analyzed data, and performed biochemical experiments, wrote and edited the manuscript; N.T., H.T.K., and M.M. designed experiments, analyzed data, wrote and edited the manuscript.

## Declaration of interests

The authors declare no competing interests.

## STAR★Methods

### Key resources table

**Table:** 

REAGENT or RESOURCE	SOURCE	IDENTIFIER
**Antibodies**		

Anti-STIM1 antibodies	Thermo Fisher Scientific	Cat# MA1-19451; RRID:AB_2197884
Anti-MTMR7 antibodies	Thermo Fisher Scientific	Cat# PA5-50342; RRID:AB_2635795

**Chemicals, peptides, and recombinant proteins**		

Fura-2 AM	Thermo Fisher Scientific	Cat# F1201
Lipofectamine 3000	Invitrogen	Cat# L3000015
Polyethyleneimine	Sigma	Cat# 306185
Thapsigargin	Thermo Fisher Scientific	Cat# T7458

**Critical commercial assays**		

QuickChange Lightning Kit	Agilent	Cat: 210518
Duolink® Proximity Ligation Assay	Millipore Sigma	DUO92002, DUO92004 DUO92008

**Deposited data**		

Raw and analyzed data	https://osf.io/ujez7/	N/A

**Experimental models: Cell lines**		

Human: HEK293T (female)	ATCC	Cat# MSPP-CRL3216)
Mouse: mouse embryonic fibroblast (MEF) cells (female) isolated from female mouse embryos harvested at E9.5	Generated in our laboratory. https://doi.org/10.1083/jcb.200102073	N/A

**Oligonucleotides**		

Primers for C338S mutant	Forward: 5′-GTGTGCTTGTTCACTCTTCTG ACGGCTGGGA-3′ Reverse: 5′-TCCCAGCCGTCAGAAGAG TGAACAAGCACAC-3′	N/A
Primers for D343A mutant .	Forward: 5′-CTGACGGCTGGGCCAG AACGGCCCA-3′ Reverse: 5′-TGGGCCGTTCTGGC CCAGCCGTCAG-3′	N/A
Primers for S569∗ mutant forward:	Forward: 5′-CTGTGGTCTGAGGTT CAGAACCCCGAG TGTTTGCT-3′ Reverse: 5′-AGCAAACACTCGGG GTTCTGAACCTCAG ACCACAG-3′	N/A

**Recombinant DNA**		

STIM1-YFP	Generated in our laboratory. https://doi.org/10.1038/embor.2011.173	N/A
MTMR7-GFP	GenScript	OMu00869C
MTMR7 without a tag	Sino Biological	MG52468
mCherry-STIM1	Addgene	#114176
SnxA-GFP	Addgene	#205128
pcDNA3.1-Akt-PH-mCherry	Addgene	#67301
PH-PLC-mCherry	Addgene	#36075
PM-GFP	Addgene	#21213
PM-FRB-mCherry	Addgene	#38004
pcDNA3.1ORAI1	Addgene	#21638

**Software and algorithms**		

Clampex 10.7 Clampfit 10.7	Molecular Devices	https://www.moleculardevices.com/
FISH-QUANT	Imbert, Arthur et al.	https://fish-quant.github.io/
ImageJ- Coloc2	Schneider et al.	https://imagej.net/plugins/coloc-2
LAS X Life Science Microscope Software Platform	Leica	https://www.leica-microsystems.com/products/microscope-software/p/leica-las-x-ls/
Volocity	Quorum Technologies	https://www.volocity4d.com
Prism	GraphPad	https://www.graphpad.com/features
SigmaPlot	Grafiti	https://grafiti.com/sigmaplot-detail/

**Other**		

PTI fluorometer	Photon Technology International	N/A
Leica TCS SP5 Laser Scanning Confocal Microscopy	Leica; University of Alberta Cell Imaging Core	N/A
ODYSSEY® XF	Licor	N/A

### Experimental model and study participant details

#### Cell culture

Mouse embryonic fibroblast (MEF) cells (female) were originally established in our laboratory from female mouse embryos harvested at E9.5, as described previously.^60^ The sex of the embryos used for MEF cell isolation does not influence the outcomes of this study. Both MEF cells and human embryonic kidney cells (HEK293T; female, ATCC Cat# MSPP-CRL3216) were cultured in accordance with established protocols using DMEM-10 (Dulbecco’s Modified Eagle’s Medium supplemented with 10% Fetal Bovine Serum, Gibco) under standard cell culture conditions of 37°C and 5% CO_2_.^61^ Cell lines used in this study were not authenticated. They were tested and are free of mycoplasma contamination. In this study, all cell lines were of female origin, which minimized potential biological variation related to sex.

#### Ethics statement

All animal-related procedures involved in MEF cells isolation adhered to the University of Alberta Animal Policy and Welfare Committee guidelines and the Canadian Council on Animal Care standards. Ethical approval for the use of mice in research was granted by the University of Alberta’s Animal Care and Use Committee for Health Sciences (Permit AUP297).

### Method details

#### Cell transfection

Transfections were performed using Lipofectamine 3000 (Invitrogen, cat# L3000015) for MEF cells and polyethyleneimine (Sigma, cat# 306185) for HEK293T cells, following the manufacturer’s instructions.^22^ For Lipofectamine 3000-mediated transfection in MEF cells, 2.5 μg plasmid DNA and 3.75 μL Lipofectamine 3000 reagent were first diluted in 2 tubes of 125 μL serum-free medium Opti-MEM (Gibco, cat#31985070), mix well and diluted with Lipofectamine 3000 reagent (1:1 ratio) followed by 15 min incubation at room temperature. DNA-lipid complex was added to cell dropwise. For polyethyleneimine-mediated transfection in HEK293T cells, plasmid DNA was diluted in Opti-MEM, and polyethyleneimine was added at a DNA:polyethyleneimine ratio of 1:3 (w/w). For a 6-well plate transfection experiment, 2 μg of plasmid DNA was mixed with 6 μg of polyethyleneimine, vortexed briefly, and incubated at room temperature for 15 min to allow formation of DNA-polyethyleneimine complexes. The mix were then added dropwise to the cells in 2 mL complete medium and gently mixed by rocking the plate.

#### Calcium measurements

MEF cells were grown to 90% confluency on the day of measurement. The cells were treated with 8 μL 1 mM Fura-2 AM in 4 mL DMEM-10 media and incubated at 37°C for 30 min. After incubation, the cells were washed with phosphate-buffered saline (PBS), replaced with 6 mL of fresh DMEM media, and incubated for an additional 15 min. Cells were then washed and harvested by trypsinization, followed by the addition of 9 mL growth media. The cell suspension was centrifuged at 2,000 rpm for 2 min, and the supernatant was discarded. The cell pellet was resuspended to a final concentration of 2x10^6^ cells/ml in 2 mL Ca^2+^-free buffer containing 20 mM HEPES, pH 7.4, 0.1% glucose, 143 mM NaCl, 6 mM KCl, 1 mM MgSO_4_.

Fluorescence measurements were performed using a PTI fluorometer (Photon Technology International). The cell suspension was transferred to a four-sided clear cuvette, and Ca^2+^ levels were monitored by fluorescence. Free cytosolic Ca^2+^ was chelated by adding 10 μL of 0.4 M EGTA at t = 1 min. Intracellular Ca^2+^ stores were depleted by adding 6 μL of 100 μM thapsigargin at t = 3 min, indirectly activating SOCE. Ca^2+^ entry via SOCE was observed by the addition of 4 μL of 1 M CaCl_2_ at t = 5 min. The maximum cytosolic Ca^2+^ level was obtained by adding 20 μL of 1 mM ionomycin and 20 μL 1 M CaCl_2_ at t = 7 min. Finally, the minimum Ca^2+^ level was determined by adding 100 μL of 0.6 M Tris, 100 μL 10%Triton X-100 and 200 μL of 0.4 M EGTA at t = 9 min Ca^2+^ measurements were performed in triplicate, and cytosolic Ca^2+^ concentration was calculated from the fluorescence ratio at 340/380 nm as described previously.^60^

#### Plasmid DNA

The primary constructs used in our studies were STIM1-YFP (yellow fluorescent protein)^62^ and MTMR7-GFP (green fluorescent protein) (GenScript ID: OMu00869C). cDNAs encoding MTMR7 mutants, C338S, D343A, and S569∗,^23^ were generated using site-directed mutagenesis (QuickChange Lightning Kit, Agilent) with a vector containing cDNA encoding mouse MTMR7 without a tag (MG52468 from Sino Biological). The oligonucleotides used for mutagenesis were as follows:

C338S: Forward: 5′-GTGTGCTTGTTCACTCTTCTGACGGCTGGGA-3’.

Reverse: 5′-TCCCAGCCGTCAGAA GAGTGAACAAGCACAC-3’.

D343A: Forward: 5′-CTGACGGCTGGGCCAGAACGGCCCA-3’.

Reverse: 5′-TGGGCCGTTCTGGCCCAGCCGTCAG-3’.

S569∗: Forward: 5′-CTGTGGTCTGAGGTTCAGAACCCCGAGTGTTTGCT-3’.

Reverse: 5′- AGCAAACACTCGGGGTTCTGAACCTCAGACCACA G-3’.

#### Immunoblot and immunoprecipitation analyses

MEF cells were harvested at 24 h after transfection followed by the addition of pre-chilled RIPA buffer containing 10 mM Tris, pH 7.4, 1 mM EDTA, 0.5 mM EGTA, 1% Triton X-100, 0.15% sodium deoxycholate, 0.1% SDS, 140 mM NaCl, and 0.1% protease inhibitor cocktail [aprotinin (0.1 mg/mL), phosphoramidone (0.1 mg/mL), TLCK (tosyl-L-lysyl-chloromethane hydrochloride, 0.1 mg/mL), TPCK (Tosylphenylalanyl chloromethyl ketone, 0.2 mg/mL), APMSF (4-(2-aminoethyl)benzenesulfonyl fluoride hydrochloride, 0.1 mg/mL), E−64 (trans-Epoxysuccinyl-L-leucylamido(4-guanidino)butane, 0.1 mg/mL), leupeptin (0.05 mg/mL), and pepstatin A (0.01 mg/mL) ]. Proteins were separated by SDS-PAGE (10% acrylamide) followed by immunoblot analysis.^63^ The protein concentration was adjusted to 1 mg/mL using 4X sample buffer (BIO-RAD, cat# 1610747) containing 10% 2-mercaptoethanol (Sigma Aldrich, cat# 21985023). Proteins were then separated by SDS-PAGE (10% acrylamide) followed by immunoblot analysis.^64^ Blots were incubated with anti-MTMR7 1:1000, anti-STIM1 1:1000 and anti-GAPDH (glyceraldehyde3-phosphate dehydrogenase) 1:2000 antibodies (Thermos Fisher #PA5-113535, # 11565-1-AP and # MAI-16757 respectively, ThermoFisher), followed by secondary antibodies (IR Dye, LI-COR). For immunoprecipitation experiments, HEK293T cells expressing STIM1-YFP or recombinant wild-type or mutant MTMR7 were harvested 48 h post-transfection followed by cross-linking and immunoprecipitation analysis.^23^

#### Proximity ligation assay

Proximity ligation assays were performed using the Duolink *In Situ* Kit (Millipore Sigma) according to the manufacturer’s instructions. MEF cells treated with or without 1 μM thapsigargin after reaching 70% confluency then fixed with 4% paraformaldehyde and permeabilized with 0.1% Triton X-100.^65^ Fixed cells were incubated with primary anti-STIM1 antibodies (1:100) and anti-MTMR7 antibodies (1:100) (Thermo Fisher cat# MA1-19451 and cat#PA5-50342) overnight at 4°C, followed by incubation with Duolink *In Situ* PLA Probe Anti-Rabbit PLUS, Duolink *In Situ* PLA Probe Anti-Mouse MINUS and Duolink *In Situ* Detection Reagents Red (Millipore Sigma), according to the manufacturer’s instructions for 1 h at 37°C. Ligation and rolling-circle amplification reactions were carried out with incubation using Duolink ligation buffer (1:5 dilution, 30 min) and amplification buffer (1:5 dilution, 100 min) at 37°C. All amplification steps were performed protected from light. The slides were mounted with Duolink *In Situ* Mounting Medium with DAPI (Millipore Sigma) and imaged using a Leica confocal microscope. DAPI was excited at 405 nm, and emission was collected between 439 and 579 nm. The proximity ligation assay signal (Red) was excited at 595 nm, with emission collected between 602 and 750 nm.

#### Fluorescence microscopy imaging

Confocal microscopy was conducted with a Leica confocal microscope equipped with a 63×/1.40 NA oil immersion objective. Mid-plane sections of MTMR7-GFP and mCherry-STIM1 expressing cells and z stack images of YFP-STIM1 expressing cells and PLA were acquired. Z-stacks were captured in *xyz* scan mode with a step size of 0.5 μm. GFP and YFP was excited at 488 nm, with emission collected from 505 to 545 nm. Excitation of mCherry was carried out at 543 nm, with emission collected between 560 and 610 nm. Quantification of STIM1 puncta was carried out using FISH-QUANT in MATLAB (https://fish-quant.github.io/). The Coloc2 plugin in ImageJ, with default parameters, was utilized to calculate Pearson’s correlation coefficients and assess co-localization between fluorescent signals.

Total Internal Reflection Fluorescence (TIRF) microscopy images were captured using Volocity software (Quorum Technologies, Canada) on a Hamamatsu EM-CDD camera (ImageEM91013, Hamamatsu Photonics, Japan) set-up on an Olympus IX-81 motorized inverted base (Evident Canada Inc.) with an 100×1.49 NA objective. GFP and mCherry were excited using laser merge module from Spectral Applied Physics (Richmond Hill, On, Canada) equipped with 491 nm, 561 solid-state lasers and through the TIRF illuminator. Wild-type and *MTMR7*^−/−^ MEF cell lines were co-transfected with plasmids encoding phosphoinositide binding domains, SnxA-GFP (Addgene #205128), pcDNA3.1-Akt-PH-mCherry (Addgene #67301), PH-PLC-mCherry (Addgene #36075) together with a generic membrane attached GFP (using the Lyn membrane targeting sequence) PM-GFP (Addgene #21213) or PM-FRB-mCherry (Addgene #38004) at a PIP reporter:membrane ratio of 2:1 using Lipofectamine 3000 according to the manufacturer’s instructions. On the day following transfection, cells were fixed with 4% paraformaldehyde, washed, and maintained in PBS during image acquisition.

Quantification of PIP levels were determined using Fiji.^66^ First, segmentation of the cell area was determined using the signal of the membrane reporter. The levels of PIPs were defined using the PIP reporter mean fluorescence intensity normalized to the membrane marker intensity. Reporter constructs were expressed at relatively low levels, and cells exhibiting excessive fluorescence or abnormal morphology were excluded from analysis. Quantitative comparisons were restricted to cells within narrow ranges of baseline reporter and membrane-marker intensity, thereby minimizing variability arising from differences in cell footprint or membrane topology. Importantly, all comparisons were performed between wild-type and *MTMR7*^−/−^ cells imaged under identical conditions, and conclusions were based on relative differences rather than absolute TIRF intensities.

#### Patch clamp analysis

Wild-type and *MTMR7*^−/−^ MEF cells were used for whole-cell patch clamp experiments. STIM-YFP, human Orai1 (pcDNA3.1; Addgene # 21638), and MTMR7-GFP (or MTMR7 mutants) or EGFP were constitutively expressed for patch clamp recordings at a 2:1:1 ratio, respectively. The Patch pipettes were prepared from soda lime capillary glass (Thermo Fisher Scientific, Waltham, MA, USA) using a Sutter P-97 puller (Sutter Instrument, Novato, CA, USA). When filled with recording solutions, pipettes had a tip resistance of 1–3 MΩ. Compositions of solutions used for whole cell patch clamp recordings of ORAI1 currents from transfected MEF cells were as follows: External (bath) solution consisted of: 5 mM HEPES, pH 7.4, 130 NaCl mM, 20 mM CaCl_2_, 4.5 mM KCl, 1 mM MgCl_2_, 10 mM D-glucose. Internal (pipette) solution consisted of: 10 mM HEPES, pH 7.2 (with CsOH), 150 mM Cs-methanesulfonate, 20 mM Cs-BAPTA, 8 mM MgCl_2_. Thapsigargin (1 μM) was added to the bath solution to pre-deplete Ca^2+^ stores prior to seal formation and break-in during whole cell patch clamp experiments. For ORAI1 currents, Ca^2+^-dependent inactivation (CDI) was evoked by 200-ms steps to −60, −80, −100 and −120 mV, delivered every 5 s from a +30-mV holding potential. For recordings of inward rectification currents, cells were depolarized to 100-mV for 10 ms followed by a ramp from +100-mV to −100 mV for 250 ms before returning to a 30-mV holding potential. A 2s interpulse was provided between sweeps. Currents were adjusted with P/6 leak subtraction during data acquisition with a 30 mV subsweep holding level. 20 mM Ca^2+^ external solution, as denoted above, was used for inward rectification currents. Recordings were filtered at 5 kHz, sampled at 10 kHz, with manual capacitance compensation and series resistance compensation between 70 and 90%, and stored directly on a computer hard drive using Clampex 10 software (Molecular Devices).^67^ Cells exhibiting a seal resistance <1GΩ following whole-cell break-in were excluded from CDI analysis. The reported “n” values in the figure legends reflect the number of recordings from individual cells from at least three independent experiments. Time constants (τ) of channel inactivation were obtained by fitting current traces with a double exponential equation in the form: (*t*) = *A*, *slow*(1−*e*^−*t*/*τ*,*slow*^) + *A*, *fast*(1−*e*^−*t*/*τ*,*fast*^) + *C*. where A(t) is the current at time t from the start of the −120 mV test pulse, A is the maximum current corresponding to either the slow or fast component, and C is a constant. The fractional fast component was calculated as: *A*,fast/*A*,*slow* + *A*,*fast* + *C*.

#### Lipid-protein interaction assay

Recombinant GST-tagged MTMR7 was incubated with Membrane Lipid Strips (P-6002, Echelon Biosciences), which contain pre-dotted spots of 15 distinct membrane lipids. Additionally, 1 μL of PI(4,5)P2 Grip and 1 μL of secondary antibody were manually spotted onto the open areas of the membrane. The membrane was then air-dried and subsequently blocked with 10 mL of PBST (PBS with 0.1% Tween 20) containing 1% BSA by gentle agitation for 1 h at room temperature. Following blocking, the membrane was incubated with 0.5 μg/mL of MTMR7-GST in 5 mL of PBST +1% BSA for 1 h. After incubation, the membrane was washed twice with 10 mL PBST for 5 min each. Primary anti-MTMR7 antibodies (1:500 dilution) were added, and the membrane was agitated for 1 h at room temperature. After washing twice with PBST for 5 min, secondary anti-mouse HRP-conjugated antibodies (1:2000 dilution) were applied, and the membrane was gently agitated for additional 30 min at room temperature. The membrane was then washed three times with 10 mL PBST for 5 min. Detection of the bound protein was performed using the LI-COR ODYSSEY imaging system.

### Quantification and statistical analysis

Image analysis was performed using Fiji/ImageJ (NIH).^66^ Quantification of proximity ligation assay (PLA) puncta and STIM1 puncta was carried out using consistent thresholding parameters across all experimental conditions. Puncta were quantified on a per-cell basis from maximum intensity projections of z stack images. Statistical analyses were performed using GraphPad Prism (GraphPad Software) except patch-clamp analysis used SigmaPlot (Systat Software). A *t* test was used to compare means between two independent groups. For comparisons involving more than two groups, one-way ANOVA followed by an appropriate post-hoc test outlined in the figure legends followed by an appropriate post-hoc test outlined in the figure legends. If the data failed a (Shapiro-Wilk) normality test, a non-parametric ANOVA on Ranks (Kruskal-Wallis) test was performed with a Dunn’s post-hoc test. Data are represented as mean ± SEM. The value of *n* represents the number of independent cells/cultures analyzed per condition, as specified in the figure legends. Statistical significance was indicated by ∗ (*p* ≤ 0.05), ∗∗ (*p* ≤ 0.01), ∗∗∗ (*p* ≤ 0.001), and ∗∗∗∗ (*p* ≤ 0.0001).
