# Supplementary material for: Localized phosphoinositide metabolism regulates STIM1/ORAI1 fast inactivation
Source: iScience. 2026 Mar 31;29(5):115543. doi: 10.1016/j.isci.2026.115543 (PMC13091563; doi:10.1016/j.isci.2026.115543)
Supplement: Document S1. Figure S1 [file mmc1.pdf]

iScience, Volume *29*

Supplemental information

Localized phosphoinositide metabolism regulates STIM1/ORAI1 fast inactivation

Ning Dai, Shawn M. Lamothe, Jody Groenendyk, Nicolas Touret, Harley T. Kurata, and Marek Michalak

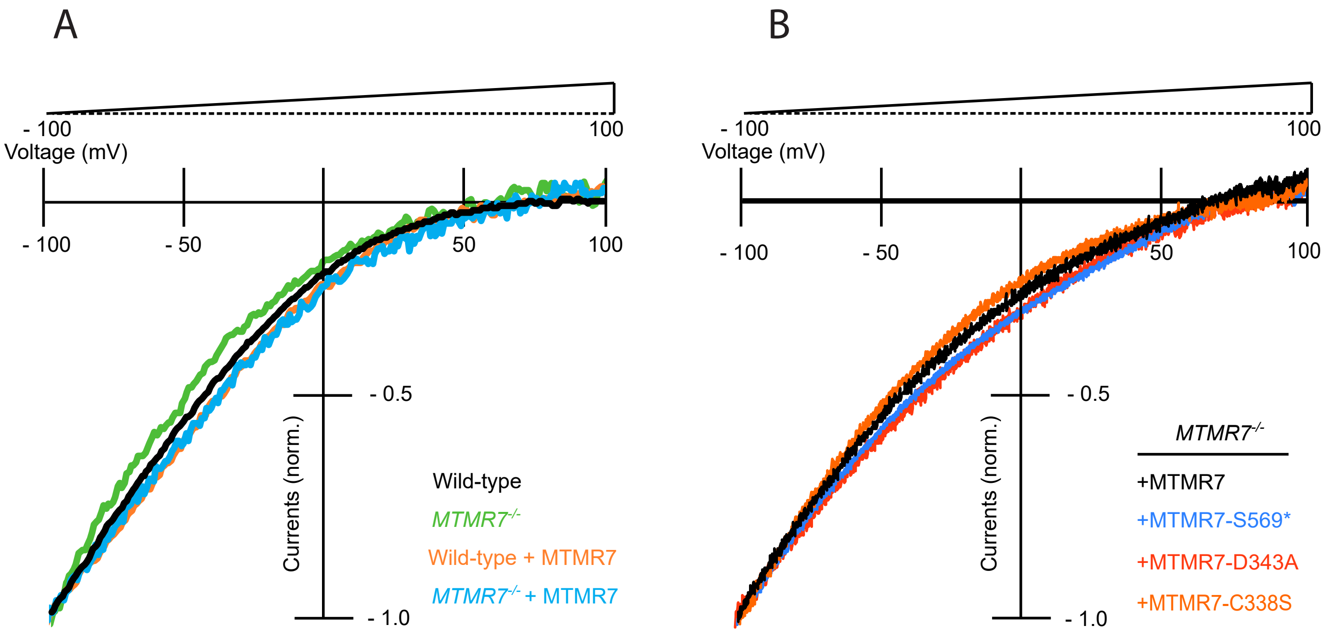

Supplemental Figure S1. Effects on ORAI1/STIM1 inward rectification.
A. Representative I-V relations are displayed as normalized currents from each group in Figure 2C. **B.** Representative I-V relations are displayed as normalized currents from each group in Figure 3F. Traces in A and B were colour matched to the corresponding groups in the Figures 2C and 3F, respectively.
